# RETRACTION: Impact of Wound Complications in Obese Versus Non‐Obese Patients Undergoing Total Hip Arthroplasty: A Meta‐Analysis

**DOI:** 10.1111/iwj.70376

**Published:** 2025-03-21

## Abstract

**RETRACTION:** YangY. , ZhaoZ. , WangY. , GaoY. , SunH. , and LiuW. , “Impact of Wound Complications in Obese Versus Non‐Obese Patients Undergoing Total Hip Arthroplasty: A Meta‐Analysis,” International Wound Journal 20, no. 10 (2023): 4200–4207, 10.1111/iwj.14318.37518969 PMC10681413

The above article, published online on 30 July 2023, in Wiley Online Library (http://onlinelibrary.wiley.com/), has been retracted by agreement between the journal Editor in Chief, Professor Keith Harding; and John Wiley & Sons Ltd. Following an investigation by the publisher, all parties have concluded that this article was accepted solely on the basis of a compromised peer review process. The editors have therefore decided to retract the article. The authors did not respond to our notice regarding the retraction.

**RETRACTION:** Y. Yang , Z. Zhao , Y. Wang , Y. Gao , H. Sun , and W. Liu , “Impact of Wound Complications in Obese Versus Non‐Obese Patients Undergoing Total Hip Arthroplasty: A Meta‐Analysis,” International Wound Journal 20, no. 10 (2023): 4200–4207, 10.1111/iwj.14318.37518969 PMC10681413

The above article, published online on 30 July 2023, in Wiley Online Library (http://onlinelibrary.wiley.com/), has been retracted by agreement between the journal Editor in Chief, Professor Keith Harding; and John Wiley & Sons Ltd. Following an investigation by the publisher, all parties have concluded that this article was accepted solely on the basis of a compromised peer review process. The editors have therefore decided to retract the article. The authors did not respond to our notice regarding the retraction.

